# Long term follow up and retrospective study on 533 gastric cancer cases

**DOI:** 10.1186/1471-2482-14-29

**Published:** 2014-05-16

**Authors:** Wei-Juan Zeng, Wen-Qin Hu, Lin-Wei Wang, Shu-Guang Yan, Jian-Ding Li, Hao-Liang Zhao, Chun-Wei Peng, Gui-Fang Yang, Yan Li

**Affiliations:** 1 Departments of Oncology & Pathology, Zhongnan Hospital of Wuhan University, Hubei Key Laboratory of Tumor Biological Behaviors & Hubei Cancer Clinical Study Center, Wuhan 430071, China; 2 Department of Surgery, Heji Hospital Affiliated to Changzhi Medical College, Changzhi 046000, China; 3 Department of Medical Imaging, The First Affiliated Hospital of Shanxi Medical University, No 85, South Jiefang Road, Taiyuan City 030001, Shangxi Province, China; 4 Department of General Surgery, Shanxi University Hospital, No 99, Longcheng Street, Taiyuan City 046000 Shangxi Province, China

**Keywords:** Gastric cancer, GC-specific overall survival, Prognosis, Multivariate analysis, Clinical pathological factors

## Abstract

**Background:**

Gastric cancer (GC) is the third leading cause of cancer death in China and the outcome of GC patients is poor. The aim of the research is to study the prognostic factors of gastric cancer patients who had curative intent or palliative resection, completed clinical database and follow-up.

**Methods:**

This retrospective study analyzed 533 GC patients from three tertiary referral teaching hospitals from January 2004 to December 2010 who had curative intent or palliative resection, complete clinical database and follow-up information. The GC-specific overall survival (OS) status was determined by the Kaplan-Meier method, and univariate analysis was conducted to identify possible factors for survival. Multivariate analysis using the Cox proportional hazard model and a forward regression procedure was conducted to define independent prognostic factors.

**Results:**

By the last follow-up, the median follow-up time of 533 GC patients was 38.6 mo (range 6.9-100.9 mo), and the median GC-specific OS was 25.3 mo (95% CI: 23.1-27.4 mo). The estimated 1-, 2-, 3- and 5-year GC-specific OS rates were 78.4%, 61.4%, 53.3% and 48.4%, respectively. Univariate analysis identified the following prognostic factors: hospital, age, gender, cancer site, surgery type, resection type, other organ resection, HIPEC, LN status, tumor invasion, distant metastases, TNM stage, postoperative SAE, systemic chemotherapy and IP chemotherapy. In multivariate analysis, seven factors were identified as independent prognostic factors for long term survival, including resection type, HIPEC, LN status, tumor invasion, distant metastases, postoperative SAE and systemic chemotherapy.

**Conclusions:**

Resection type, HIPEC, postoperative SAE and systemic chemotherapy are four independent prognostic factors that could be intervened for GC patients for improving survival.

## Background

Gastric cancer (GC) remains the second leading cause of cancer death worldwide [1], accounting for 8% of the total cases and 10% of total deaths in 2008 [2]. In China, GC is the third leading cause of cancer death [3] and the outcome of GC patients is poor, especially for patients at advanced stage, and the 5-year survival rate is less than 20%-25% [4].

Early diagnosis and early treatment remain the best strategy for GC. In China, however, a majority of GC patients are not early cancer by the time when they seek medical attention [5,6]. Therefore, surgery-based multidisciplinary treatment approach is warranted in order to improve both overall survival (OS) and the quality of life.

Despite this common-sense knowledge, there is no commonly accepted multidisciplinary treatment strategy in China, primarily due to the lack of large database information reflecting the clinical reality of the current treatment situation.

In our previous studies on GC patients, we evaluated the common tumor markers for the diagnosis of gastric cancer. In these relatively large cohort studies, stage III and beyond patients accounted for over 65% of the entire patient population [6,7], a result similar to other reports from China [5,8]. For these patients, GC is no longer a local disease, but at least a regional or a systemic disease.

Currently, surgery remains the most effective therapy for GC, offering an excellent chance (90%) of a cure for early GC patients [9]. Surgical procedures have a big impact on OS and recurrence [10]. R0 resection with D2 lymphadenectomy is regarded as the standard surgical technique [11,12], as D2 lymphadenectomy had lower recurrence and GC-related death rates [13]. However, for stage III and beyond patients, the currently adopted surgical procedure only removes local tumor mass but often neglects the micro-metastases. Therefore, additional adjuvant therapies are required to ensure better treatment efficacy.

Over the past years, our database has grown bigger and more detailed information on major clinico-pathological characteristics has been accumulated. Therefore, we conducted this comprehensive analysis of the data collected from three major teaching hospitals in Central China, so as to gain deeper insights to the major features of GC in central China and to identify independent factors for prognosis that could be intervened.

## Methods

### Ethics statement

All patients provided written informed consent for their information to be stored in the hospital database; and we obtained separate consent for research. Study approval was obtained from independent ethics committees from Zhongnan Hospital of Wuhan University. The study was undertaken in accordance with the ethical standards of the World Medical Association Declaration of Helsinki.

### Patients

This study included a total of 533 GC patients from three tertiary referral hospitals, from January 2004 to December 2010. These patients underwent resection with curative intent (D2 lymphadenectomy) or palliative resection. All the detailed clinic-pathological information was available, including demographic variables, underlying co-morbidities, surgical modality, lab and image study information, pathological reports, pre- and post-operative therapies, and follow-up information. Pathological information was mainly focused on tumor type, pathological grading, TNM stages, blood vessel or neural invasions. The pathologic staging was based on the 7th edition of AJCC staging criteria [14]. Postoperative treatments were focused on chemotherapy regimens and cycles, and radiotherapy if applicable. GC patients with T2 or higher, any N tumors should receive systemic chemotherapy except patients who declined the offer [15]. Hyperthermic intraperitoneal chemotherapy (HIPEC) and intraperitoneal chemotherapy (IP chemotherapy) were adjuvant chemotherapy, and only those who had peritoneal carcinomatosis (PC) should receive [16]. In our study, the systemic chemotherapy administered were mainly FOLFOX4 and FOLFOX6, HIPEC were mainly using lobaplatin and paclitaxel, and IP chemotherapy were docetaxel and carboplatin.

These patients were followed-up every 3 months during the first 2 years after operation, every 6 months on the third postoperative year and every year thereafter. All the follow-up information was incorporated into a standardized database.

### Database construction

The above-mentioned information was incorporated into a central database, set up at the Zhongnan Hospital of Wuhan University, which undergoes regular updating every 3 months.

### Statistical analysis

All eligibility cases from the central database were analyzed by SPSS 17.0 statistical package software (SPSS Inc., Chicago, IL, USA). The variables were hospital (Zhongnan Hospital, Heji Hospital or Hubei Tumor Hospital), gender (male or female), age (≤65 yr or > 65 yr), cancer site (upper third [excluding squamous cell carcinoma at gastroesophageal junction], middle third, lower third or whole stomach), pathological type (well or intermediately differentiated adenocarcinoma, poorly differentiated or undifferentiated carcinoma, signet ring cell carcinoma or mucious adenocarcinoma or others), surgery type (proximal gastrectomy, distal gastrectomy or total gastrectomy), resection type (for stomach itself) (palliative resection or curative resection), other organ resection (mainly included liver, spleen, intestines, ovarian, ovarian ducts) (0, 1, 2 or ≥ 3), HIPEC (yes or no), lymph node status (LN status) (N0, N1, N2 or N3), tumor invasion (T1, T2, T3, T4a or T4b), distant metastasis (M0 or M1), pathological stage (I, II, IIIA, IIIB, IIIC or IV) [14], postoperative serious adverse event (postoperative SAE) (defined as life threatening events after operation, including gastrointestinal obstruction, anastomotic leakage, and bleeding leading to grade 3 and above anemia, abdominal abscess) (yes or no), systemic chemotherapy (0, 1 to 6 cycles or > 6 cycles), IP chemotherapy (yes or no), GC-specific overall survival (GC-specific OS, defined as the time interval from first treatment to GC-specific death, with the last follow-up time on May 31, 2012).

The numerical data was analyzed directly. The category data was converted when necessary. The Kaplan-Meier survival curve was used to study the survival status, using log rank test to decipher the statistical significance, which was judged as *P* < 0.05 throughout this study.

To work out independent factors for survival, a Cox proportional hazard model was used to first obtain the possible factors and then used forward regression procedure to finally identify the independent factors.

## Results

### Characteristics of the patients

A total of 533 patients with GC were recruited from 3 tertiary referral teaching hospitals, including 194 patients from Zhongnan Hospital of Wuhan University, 182 patients from Heji Hospital and 157 patients from Hubei Tumor Hospital. By the time of last follow-up, 278 deaths (52.2%) occurred, including 126 deaths (64.9%) out of 194 enrolled patients from Zhongnan Hospital of Wuhan University, 84 deaths (46.2%) out of 182 enrolled patients from Heji Hospital, and 68 deaths (43.3%) out of 157 enrolled patients from Hubei Tumor Hospital. The median age of cases was 58 years (range 20–85 years), and male-to-female ratio was 2.7 to 1. Detailed information on major demographic and clinico-pathological characteristics was listed in Table 1.

**Table 1:** Characteristics of the 533 GC patients enrolled into this study

**Variables**	**Total n (%)**	**Events n (%)**	**Median GC-specific OS (95% CI) (mo)**	***P *** **value**
Age (yr)				
≤ 65	380 (71.3)	178 (46.8)	51.7 (39.7-63.7)	**< 0.001**
> 65	153 (28.7)	100 (65.4)	28.0 (21.6-34.4)	
Gender				
Male	389 (73.0)	192 (49.4)	39.7 (29.9-49.5)	**0.019**
Female	144 (27.0)	86 (59.7)	28.0 (17.9-38.1)	
Cancer site				
Upper third	156 (29.3)	80 (51.3)	32.6 (25.9-39.3)	**0.004**
Middle third	119 (22.3)	61 (51.3)	38.9 (9.4-68.4)	
Lower third	222 (41.7)	112 (50.5)	42.1 (34.2-49.9)	
Whole stomach	36 (6.8)	25 (69.4)	13.2 (10.1-16.3)	
Pathological type				
Adeno WD/ID	131 (24.6)	59 (45.0)	42.1 (29.9-54.2)	0.212
Adeno PD/UN	299 (56.1)	160 (53.5)	34.9 (27.5-42.4)	
Signet ring/mucious Ca	85 (15.9)	49 (57.6)	28.0 (10.9-45.1)	
Others	18 (3.4)	10 (55.6)	33.7 (20.0-47.5)	
Surgery type				
Proximal gastrectomy	169 (31.7)	82 (48.5)	35.9 (20.5-51.3)	**< 0.001**
Distal gastrectomy	268 (50.3)	128 (47.8)	46.6 (38.1-55.1)	
Total gastrectomy	96 (18.0)	68 (70.8)	17.4 (11.3-23.4)	
Resection type				
Palliative resection	11 (2.1)	11 (100.0)	9.8 (8.0-11.6)	**< 0.001**
Curative resection	522 (97.9)	267 (51.1)	38.9 (31.8-46.0)	
Other organ resection (n)				
0	507 (95.1)	256(50.5)	39.3 (32.5-46.0)	**< 0.001**
1	14 (2.6)	11 (78.6)	24.1 (9.6-38.7)	
2	8 (1.5)	7 (87.5)	12.4 (2.7-22.2)	
≥ 3	4 (0.8)	4 (100.0)	13.6 (2.7-24.4)	
HIPEC				
No	505 (94.7)	251 (49.7)	39.7 (32.4-47.0)	**< 0.001**
Yes	28 (5.3)	27 (96.4)	13.4 (9.6-17.2)	
LN status				
N0	172 (32.3)	51 (29.7)	67.3 (59.8-74.8)	**< 0.001**
N1	112 (21.0)	57 (50.9)	35.9 (26.8-45.0)	
N2	143 (26.8)	86 (60.1)	27.0 (19.9-34.1)	
N3	106 (20.0)	84 (30.5)	14.4 (12.0-16.8)	
Tumor invasion				
T1	25 (4.7)	3 (12.0)	75.4 (66.4-84.4)	**< 0.001**
T2	85 (15.9)	19 (22.4)	72.7 (62.2-83.2)	
T3	2 (0.4)	1 (50.0)	29.1 (10.7-47.4)	
T4a	332 (62.3)	187 (56.3)	33.0 (26.6-39.4)	
T4b	89 (16.7)	68 (76.4)	14.8 (10.8-18.9)	
Distant metastases				
No	478 (89.7)	224 (46.9)	42.5 (34.6-50.4)	**< 0.001**
Yes	55 (10.3)	54 (98.2)	10.6 (9.0-12.1)	
TNM staging				
Stage I	79 (14.8)	8 (10.1)	85.2 (76.1-94.3)	**< 0.001**
Stage II	100 (18.8)	35 (35.0)	53.9 (46.6-61.3)	
Stage IIIA	80 (15.0)	38 (47.5)	40.0 (21.7-58.3)	
Stage IIIB	116 (21.8)	67 (57.8)	28.0 (14.9-41.1)	
Stage IIIC	117 (22.0)	90 (76.9)	14.8 (10.6-19.1)	
Stage IV	41 (7.7)	40 (97.6)	11.1 (9.7-12.4)	
Postoperative SAE				
No	458 (85.9)	205 (44.8)	49.8 (32.5-67.0)	**< 0.001**
Yes	75 (14.1)	73 (97.3)	14.8 (10.0-19.6)	
Systemic chemotherapy (cycles)				
0	217 (40.7)	128 (59.0)	26.3 (19.2-33.4)	**0.001**
1 to 6	302 (56.7)	142 (47.0)	51.7 (36.6-66.9)	
> 6	14 (2.6)	8 (57.1)	37.8 (16.9-58.7)	
IP chemotherapy				
No	521 (97.7)	267 (51.2)	37.0 (29.8-44.2)	**0.003**
Yes	12 (2.3)	11 (91.7)	11.1 (7.0-15.1)	

GC: gastric cancer; GC-specific OS: gastric cancer-specific overall survival; Adeno WD/ID: well differentiated or intermediately differentiated adenocarcinoma; Adeno PD/UN: poorly differentiated or undifferentiated carinoma; Signet ring/mucious Ca: Signet ring cell carcinoma or mucious adenocarcinoma; HIPEC: hyperthermic intraperitoneal chemotherapy; LN status: lymph node status; SAE: serious adverse event; IP chemotherapy: intraperitoneal chemotherapy.

### GC-specific OS

**Figure 1:**
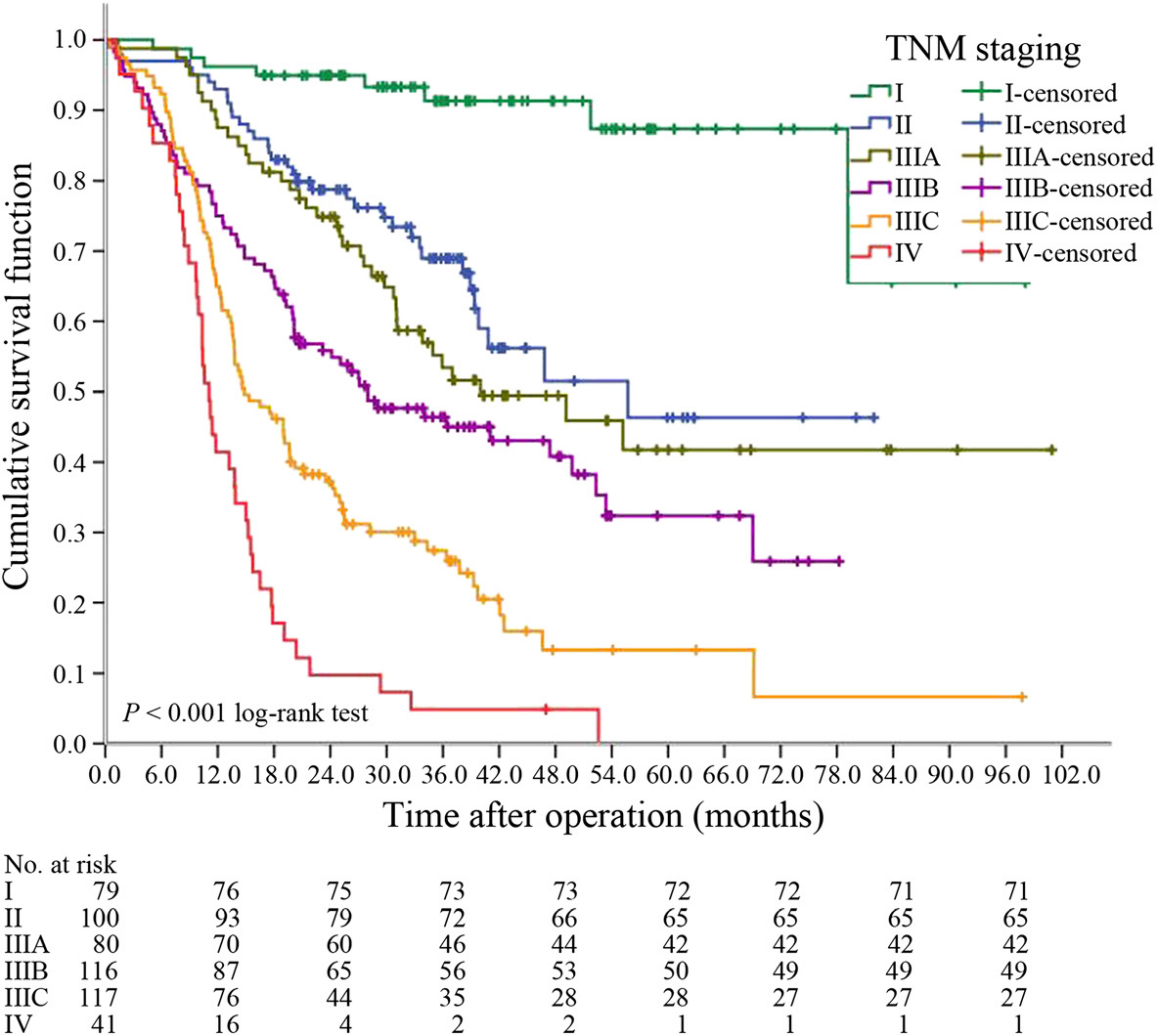
Kaplan-Meier survival curve of the 533 GC patients in this study.

By the time of last follow-up, the median follow-up time was 38.6 mo (range 6.9-100.9 mo), and 278 patients died out of the entire 533 assessable patients (52.2%). The median GC-specific OS was 25.3 mo (95% CI: 23.1-27.4 mo). The survival curve by stages was shown in Figure 1. The estimated 1-, 2-, 3- and 5-year GC-specific OS rates were 78.4%, 61.4%, 53.3% and 48.4%, respectively. The median survival by stages I, II, IIIA, IIIB, IIIC and IV were 85.2 mo (95% CI: 76.1-94.3 mo), 53.9 mo (95% CI: 46.6-61.3 mo), 40.0 mo (95% CI: 21.7-58.3 mo), 28.0 mo (95% CI: 14.9-41.1 mo), 14.8 mo (95% CI: 10.6-19.1 mo) and 11.1 mo (95% CI: 9.7-12.4 mo), respectively. As shown in Figure 1, significant differences in GC-specific OS were found among different clinical stages. Patients at clinical stage IIIB and beyond had much poorer GC-specific OS status than other patients.

### Mortality analysis

**Figure 2:**
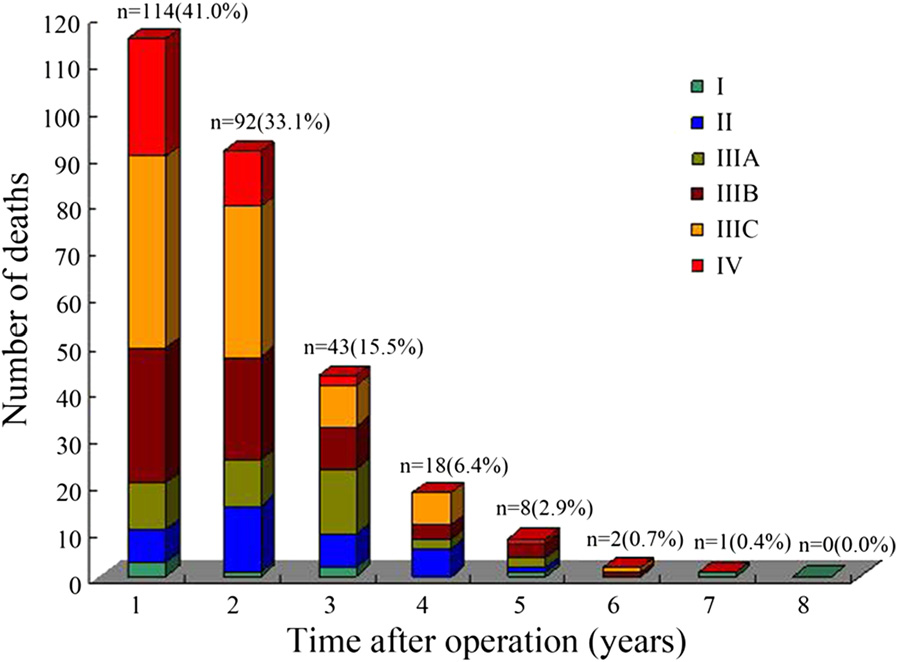
Information on GC-specific deaths in relationship with clinical stages.

By the time of last follow-up, 278 patients (52.2%) died among the entire 533 assessable patients. In terms of absolute number of patient death on the yearly basis, there were 114 (41.0%), 92 (33.1%), 43 (15.5%), 18 (6.5%), 8 (2.9%) deaths, respectively, in the 1st, 2ed, 3rd, 4th, and 5th postoperative year. Only 3 (1.1%) deaths occurred after 5 years. Information on GC-specific death in relationship with clinical stages was depicted in Figure 2. Putting together, there were 249 (89.6%) deaths within three years after operation.

### Univariate survival analysis

In this study, all variables were analyzed by Kaplan-Meier curve and log-rank test. Among these variables, pathological type had no statistically significant impact on GC-specific OS (*P* = 0.212), but statistically significant factors were hospital (*P* = 0.008), age (*P* < 0.001), gender (*P* = 0.019), cancer site (*P* = 0.004), surgery type (*P* < 0.001), resection type (*P* < 0.001), other organ resection (*P* < 0.001), HIPEC (*P* < 0.001), LN status (*P* < 0.001), tumor invasion (*P* < 0.001), distant metastases (*P* < 0.001), TNM stage (*P* < 0.001), postoperative SAE (*P* < 0.001), systemic chemotherapy (*P* = 0.001), and IP chemotherapy (*P* = 0.003) (Table 1).

### Multivariate survival analysis

After univariate survival analysis, the above significant factors were further subjected to multivariate analysis using Cox proportional hazard model and forward regression procedure. The following variables were identified as independent factors for prognosis: tumor invasion (*P* < 0.001), LN status (*P* < 0.001), distant metastases (*P* < 0.001), resection type (*P* = 0.015), HIPEC (*P* = 0.049), postoperative SAE (*P* < 0.001) and systemic chemotherapy (*P* < 0.001) (Table 2).

**Table 2:** Independent prognostic factors of 533 GC patients identified by multivariate analysis

**Covariate**	**χ**^**2**^	***P***	**HR**	**95% CI**	
				**Lower**	**Upper**
Tumor invasion	13.008	< 0.001	1.022	1.010	1.034
LN status	36.845	< 0.001	1.462	1.293	1.653
Distant metastases	29.004	< 0.001	2.832	1.939	4.137
Resection type	5.900	0.015	0.430	0.218	0.850
HIPEC	3.863	0.049	1.707	1.001	2.910
Postoperative SAE	27.752	< 0.001	2.507	1.781	3.528
Systemic chemotherapy	24.064	< 0.001	0.521	0.402	0.676

GC: gastric cancer; HR: hazard ratio; CI: confidence interval; LN status: lymph node status; HIPEC: hyperthermic intraperitoneal chemotherapy; SAE: serious adverse event.

## Discussion

Several important points should be considered from this study. First, a majority of GC patients are at advanced clinical stage. In our series of 533 patients, 354 cases (66.4%) were clinically stage III and beyond. For these patients, GC is no longer a local disease, but at least a regional or a systemic disease. Although surgery could remove the bulky tumor mass itself, it may leave some unseen cancer cells in the operating field. Therefore, more intensive adjuvant chemotherapy should be followed in order to eradicate these left-over cancer cells. Two large scale randomized clinical trials have already demonstrated the superiority of this approach over conventional surgery alone [17,18]. Another reasonable approach is to start perioperative chemotherapy, to down-stage the tumor, followed by curative resection. It has been proven that such a treat modality indeed could improve the clinical outcomes of GC patients [19].

Secondly, our analysis found that over 40% of GC death occurred in the first year after operation, and another 30% plus of GC death occurred during the second year after operation [20-22]. Therefore, it is clinically important to design rational strategies to address these problems. One key consideration is that high risk factors should be investigated and identified, so as to reduce them and reduce the death risk. Another strategy is to design a close follow-up plan and strictly implement it, so as to identify those patients with early signs of recurrence and apply appropriate therapies. Among the currently used methods, serum tumor markers study and medical imaging studies are most widely used approaches. Regular monitoring blood tumor markers carcinoembryonic antigen (CEA) and carboxyl antigen 19–9 (CA19-9) could help provide warning information on cancer recurrence [23].

Various clinicopathological factors have been reported to impact on GC-specific OS, such as age, gender, cancer site, surgery type, resection type, other organ resection, HIPEC, LN status, tumor invasion, distant metastases, TNM stage, postoperative SAE, systemic chemotherapy and IP chemotherapy [5,7,8,20,24-28]. These results are in accordance with our study. In our study, pathological type had no impact on GC-specific OS, which is not in conformity with several previous studies that concluded that pathological type was an important factor for prognosis and survival of GC [5,24], but is in agreement with some other studies that reported that pathological type had no influence on GC-specific OS [7,27]. This could be due to the different typing method used. It has been documented that Lauren histological classification is a simple and practical typing method to have significant correlation with survival of GC. Clinicalpathological information of this study was obtained from hospital department of pathology, and they did not adopt Lauren classification. In future studies, we should adopt this classification.

For cancer patients, the clinical outcomes depend on several important factors, which could be divided into those that cannot be intervened such as TNM stage, and those can be intervened such as treatment models. After the Cox proportional hazard model analysis, we worked out seven independent factors that had significant impact on survival, six of these seven factors have already been well recognized as the most important determinants of patients’ survival [5,7,8,29]. What deserves special attention is the finding that HIPEC is also an independent factor for improved survival. Several phases I to III studies have already demonstrated the treatment advantage of HIPEC. Glehen et al. consecutively treated 49 advanced GC patients with HIPEC, which resulted in 10.3 months of GC-specific OS, against 6.1 months of GC-specific OS treated with only standard curative resection [30]. In another study by Yonemura et al., 107 GC patients also treated with HIPEC, and the GC-specific OS was 11.5 months [31]. More importantly, a recent phase III prospective randomized clinical trial also confirmed the survival advantage of 11.0 months in the HIPEC group against 6.5 months in the CRS group [32]. In addition, a systematic review and meta-analysis of 13 acceptable qualities randomized controlled trials also have established that HIPEC has significant survival advantage over the currently standard treatment for advanced GC [33]. Taken together, all these facts confirm the value of HIPEC for the treatment of stage III and beyond GC patients. In this study, the median survival of patients with HIPEC was 13.4 mo (95% CI: 9.6-17.2), which was shorter than others without HIPEC (39.7 mo [95% CI: 32.4-47.0]). It was due to patients with HIPEC were gastric cancer with metastasis and prognosis was not optimistic. However, the median survival of patients with HIPEC was longer than patients with metastasis (13.4 mo *vs* 10.6 mo, *P* < 0.05). It supports HIPEC has significant survival advantage even though there could be selection bias in this regard, due to the limited number of patients treated by HIPEC.

Postoperative SAE included gastrointestinal obstruction, anastomotic leakage, and bleeding leading to grade 3 and above anemia, abdominal abscess. All these have been confirmed to have a significant negative impact on GC-specific OS. In the study of Sierzega et al. [34], the median OS of patients with anastomotic leakage was significantly lower than patients with non-anastomotic leakage (4.1 mo vs. 23 mo, *P* < 0.001), and the progression-free survival of patients with anastomotic leakage was also significantly shorter than patients with non-anastomotic leakage (11 mo vs. 19 mo, *P* = 0.021). In another study by Yoo et al. [35], the mean OS of patients with anastomotic leakage was significantly lower than patients with non-anastomotic leakage (30.5 mo vs. 96.2 mo, *P* < 0.001). Anastomotic leakage could promote gastric cancer progression by prolonging inflammation [34-36]. According to Tokunaga et al. [37], GC patients with intra-abdominal infection had a poorer 5-year OS rate and 5-relapse-free survival rate than patients without intra-abdominal infectins (66.4% vs. 86.8%, *P* < 0.001 and 64.9% vs. 84.5%, *P* < 0.001). In another study by Li et al. [38], postoperative complications including gastrointestinal obstruction, anastomotic leakage, and bleeding leading and abdominal abscess all were independent and negative prognostic factors for GC. Therefore, all efforts should be made to reduce the risk for postoperative SAE, including careful patient selection for surgery and optimized perioperative patient care.

## Conclusion

In summary, this study identified four independent prognostic factors that could be intervened for GC patients, including curative resection, HIPEC, postoperative SAE and systemic chemotherapy, and three independent prognostic factors that cannot be intervened: tumor invasion, LN status and distant metastasis. Therefore, increasing attention should be directed at better understanding tumor biology involved in cancer invasion and metastasis, and refining multi-disciplinary comprehensive treatment strategies to enhance efficacy and reduce SAE.

## Competing interests

The authors declare that they have no competing interests.

## Authors’ contributions

YL, GFY and JDL conceived of the study, and participated in its design and coordination. WQH, LWW, SGY, HLZ, CWP and WJZ participated in the database collection and follow-up. WJZ performed the statistical analyses and drafted the manuscript. All authors read and approved the final manuscript.

## Pre-publication history

The pre-publication history for this paper can be accessed here:

http://www.biomedcentral.com/1471-2482/14/29/prepub
